# Deletion of murine *slc29a4* modifies vascular responses to adenosine and 5‐hydroxytryptamine in a sexually dimorphic manner

**DOI:** 10.14814/phy2.14395

**Published:** 2020-03-13

**Authors:** Ran Wei, Stephen L. Gust, David Tandio, Alexia Maheux, Khanh H. Nguyen, Joanne Wang, Stephane Bourque, Frances Plane, James R. Hammond

**Affiliations:** ^1^ Department of Pharmacology University of Alberta Edmonton AB Canada; ^2^ Department of Pharmaceutics University of Washington Seattle WA USA; ^3^ Department of Anaesthesia and Pain Medicine University of Alberta Edmonton AB Canada

**Keywords:** 5‐HT, adenosine, mesenteric artery, transporters, vascular

## Abstract

Equilibrative nucleoside transporter 4 (ENT4), encoded by *SLC29A4*, mediates the flux of both 5‐hydroxytryptamine (5‐HT) and adenosine across cell membranes. We hypothesized that loss of ENT4 function in mice would modify the effects of these established regulators of vascular function. Male and female wild‐type (WT) and *slc29a4*‐null (ENT4‐KO) mice were compared with respect to their hemodynamics and mesenteric vascular function. Male ENT4‐KO mice had a complete loss of myogenic tone in their mesenteric resistance arteries. This was accompanied by a decrease in blood flow in the superior mesenteric artery in the male ENT4‐KO mice, and a reduced responsiveness to 5‐HT. In contrast, endothelium‐dependent relaxations of mesenteric arteries from female ENT4‐KO mice were more sensitive to Ca^2+^‐activated K^+^ (K_Ca_) channel blockade than WT mice. Female ENT4‐KO mice also demonstrated an enhanced vasodilatory response to adenosine in vivo that was not seen in males. Ketanserin (5‐HT_2A_ inhibitor) and GR55562 (5‐HT_1B/1D_ inhibitor) decreased 5‐HT‐induced tone, but only ketanserin inhibited the relaxant effect of 5‐HT in mesenteric arteries. 5‐HT‐evoked increases in tone were elevated in arteries from ENT4‐KO mice upon block of endothelial relaxant pathways, with arteries from female ENT4‐KO mice showing the greatest increase. Adenosine A_2b_ receptor expression was decreased, while other adenosine transporter subtypes, as well as adenosine deaminase and adenosine kinase were increased in mesenteric arteries from male, but not female, ENT4‐KO mice. These findings indicate that deletion of *slc29a4* leads to sex‐specific changes in vascular function with significant consequences for regulation of blood flow and pressure by adenosine and 5‐HT.

## Graphical abstract

Loss of the adenosine/5‐HT transporter ENT4 (encoded by slc29a4) leads to sex‐specific changes in vascular regulation in the ENT4‐null mouse. Male mice have a complete loss of myogenic tone in their mesenteric arteries, while mesenteric arteries from female mice exhibit a change in the relative contributions of nitric oxide and KCa channels to vascular regulation. The vascular effects of both adenosine and 5‐HT are enhanced in slc29a4‐null mice. These data indicate that ENT4 plays an important role in the modulation of adenosine and 5‐HT activity in normal vascular function.

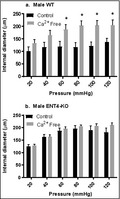

## 1 INTRODUCTION

*SLC29A4* encodes for an integral plasma membrane protein known as PMAT (Plasma Membrane Monoamine Transporter) or ENT4 (Equilibrative Nucleoside Transporter 4). ENT4 differs from ENTs encoded by other members of the *SLC29* family (ENT1, ENT2, ENT3) in that it also transports monoamines such as 5‐hydroxytryptamine (5‐HT; Engel, Zhou, & Wang, 2004; Shirasaka et al., 2017; Wang, 2016). Furthermore, it has a more restricted specificity for nucleosides than other ENTs, accepting only adenosine and some adenosine analogues (Tandio, Vilas, & Hammond, 2019) as substrates. There have been numerous studies highlighting the role of ENT4 in modulating the effects of 5‐HT in the CNS, but relatively few studies have examined its role in peripheral systems. Since both adenosine and 5‐HT have well‐documented cardiovascular effects (Fidalgo, Ivanov, & Wood, 2013; McIntosh & Lasley, 2012; Sousa & Diniz, 2017; Watts, 2016), ENT4 may play a role in regulating the effects of these agents in the vasculature. A unique aspect of ENT4 is the enhancement of substrate flux in acidic conditions, such as those associated with vascular ischemia‐reperfusion injury (Barnes et al., 2006; Tandio et al., 2019; Zhou, Duan, Engel, Xia, & Wang, 2010). ENT4 has been implicated in regulating 5‐HT levels in rat heart, particularly during the reperfusion stage of ischemia‐reperfusion (Sonobe, Akiyama, Du, & Pearson, 2019), and modulation of adenosine actions in the vasculature has long been proposed as a therapy to attenuate ischemia‐reperfusion injury (Abd‐Elfattah, Aly, Hanan, & Wechsler, 2012; Abd‐Elfattah et al., 2013; Hirai & Ashraf, 1998; Rose et al., 2010; Van Belle, 1995; Yang & Leung, 2015). Therefore, ENT4 may prove to be a novel drug target for therapeutic intervention in ischemia‐reperfusion injury.

Adenosine, often referred to as a ‘retaliatory metabolite’, is released from cells during periods of cell stress and acts on extracellular receptors to mediate protective actions such as vasodilation, anti‐inflammatory, angiogenic, and anti‐thrombotic effects (Newby, 1984). The mechanisms underlying the cardiovascular effects of 5‐HT are less well defined. 5‐HT has been shown to dilate some isolated vessels whereas in others it causes constriction or produces a concentration‐dependent biphasic response (Calabrese, 2001; Kaumann & Levy, 2006; Watts, Morrison, Davis, & Barman, 2012). This has been attributed to the relative expression of 5‐HT receptor subtypes in different tissues and species (Watts et al., 2012). In mice, the species under consideration in the present study, 5‐HT has been reported to have a contractile effect on isolated arteries (Islam et al., 2014; Matsumoto, Kobayashi, Ishida, Taguchi, & Kamata, 2010; McKune & Watts, 2001). The in vivo vascular response to 5‐HT, on the other hand, is typically vasodilation, reflecting an interplay between its direct vascular actions and its involvement in the neural regulation of cardiovascular function (Watts et al., 2012). We thus hypothesized that, if ENT4 was playing a significant role in the regulation of 5‐HT and/or adenosine levels in the vasculature, then changes in ENT4 activity would have a significant impact on vascular regulation by these agents. In particular, loss/inhibition of ENT4 is anticipated to lead to increases in extracellular adenosine, as has been seen in ENT1‐null mice (Best, Bone, Vilas, Gros, & Hammond, 2018; Warraich et al., 2013), and extracellular 5‐HT, as has been reported for SERT‐null mice (Li, 2006), and thereby increase their vascular activities via enhanced stimulation of their respective extracellular receptors. We tested this hypothesis by examining the vascular reactivity of the *slc29a4*‐null (ENT4‐KO) mouse. We (a) evaluated differences in blood pressure and mesenteric artery flow in vivo through the use of indwelling carotid pressure sensors and mesenteric flow monitors in anesthetized mice, (b) compared the myogenic reactivity of second‐order mesenteric arteries from wildtype (WT) and ENT4‐KO mice, (c) assessed the ability of mesenteric arteries from WT and ENT4‐KO mice to respond to relaxing (acetylcholine, sodium nitroprusside (SNP), adenosine) and vasoconstrictor (phenylephrine, 5‐HT) stimuli, and (d) determined the relative role of endothelium‐derived nitric oxide (NO) and endothelial Ca^2+^‐activated K^+^ (K_Ca_) channels in mediating the acetylcholine‐directed endothelium‐dependent relaxation in mesenteric vessels from WT and ENT4‐KO mice. In addition, potential compensatory changes in other components of the adenosine and 5‐HT signaling and metabolic pathways in the ENT4‐KO mesenteric artery were assessed by qPCR. Our data describe significant effects of *slc29a4*/ENT4 deletion on vascular regulation, and a clear sexual dimorphism with respect to modifications in myogenic tone and the relative contribution of endothelium‐derived NO and K_Ca_ channels to this regulation.

## 2 MATERIALS AND METHODS

### 2.1 Animals

Mice (C57BL/6J background) with targeted disruption of *slc29a4* (global knockout) were generously donated by Dr. Joanne Wang (Duan & Wang, 2013). Thereafter, all mice were bred in house, via homozygous mating pairs, using standard husbandry procedures. Mice were used for experiments at 12–16 weeks of age. For tissue collection, mice were euthanized by isoflurane inhalation followed by decapitation according to the standards of the Canadian Council on Animal Care and protocols approved by the Animal Care Committee of the Faculty of Medicine & Dentistry, University of Alberta.

### 2.2 Hemodynamic analyses

Adult mice (~12 weeks of age) were anesthetized with isoflurane (3%–5% in 100% O_2_) and kept on a warming platform connected to a circulating water bath; a rectal thermometer was used to monitor body temperature throughout. Mice were tracheotomized and mechanically ventilated and then instrumented with a fibre‐optic pressure sensor (FISO Technologies Inc.) in the right carotid artery for blood pressure and heart rate monitoring. Silastic catheters (Micro‐renathane – 0.010” o.d., 0.005” i.d.) were inserted into the left and right femoral veins for fluid and drug delivery. Perivascular blood flow probes (0.5 PSL precision nanoprobes; Transonic Systems Inc.) were then placed around the superior mesenteric artery to measure regional blood flow to the gut. Circulatory parameters (including blood pressure, heart rate, blood flow measurements) and vital signs (body temperature, oxygen saturation, respiratory rate, expired carbon dioxide) were monitored continuously. To ensure stable hemodynamic parameters, blood pressure was allowed to equilibrate for 25 min after cannulation, after which time baseline values were collected for 15 min. After baseline recordings, hemodynamic responses to methacholine (1 µg/kg), 5‐HT (50 µg/kg), adenosine (100 mg/kg), or L‐nitroarginine methyl ester (L‐NAME; 60 mg/kg) (all dissolved in saline and injected as a single bolus dose at a volume of 1 ml/kg) were assessed. The concentrations of methacholine and 5‐HT were selected by titration as the maximum dose that affected blood pressure with no direct effect on heart rate, suggesting a predominantly vascular effect on blood pressure.

### 2.3 Pressure myography

The mesenteric vascular bed was removed and placed into ice‐cold Krebs buffer containing (mM): NaCl 119.0, NaHCO_3_ 25.0, KCl 4.7, MgSO_4_ 1.2, KH_2_PO_4_ 1.18, glucose 11, disodium EDTA 0.027 and CaCl_2_ 2.5. Second‐order mesenteric arteries were removed and cleaned of adhering fat and connective tissue. Leak‐free segments of second‐order mesenteric arteries (2–3 mm) were mounted in a pressure myograph as previously described (Doughty, Plane, & Langton, 1999). Briefly, vessels were mounted between two glass cannulae in an arteriograph chamber (Living Systems Instrumentation) filled with Krebs' solution and secured with thin monofilament sutures. The arteriograph was placed on the stage of an inverted microscope (Eclipse TE300; Nikon) and connected to a peristaltic pump regulated by a pressure servo‐controller (Living Systems Instrumentation). Images of the vessel were captured using a Sony XC‐73CE monochrome camera module and measurement of arterial diameter was via an automated video dimension analyzer (Living Systems Instrumentation). Vessel diameters and pressure measurements were recorded via a PowerLab acquisition system using Chart 5.0 software (AD Instruments). Vessels were bathed in Krebs’ buffer gassed with 95% O_2_/5% CO_2_ at 37°C, and intravascular pressure was maintained via a pressure servo‐control system. Following a 30 min equilibration period with intravascular pressure set at 80 mmHg, a pressure ramp was applied by increasing the pressure from 20 mmHg to 120 mmHg in increments of 20 mmHg. Each pressure step was held for 2–3 min or until the diameter stabilized. Arteries were then bathed in zero Ca^2+^ Krebs solution and allowed to reach the maximum passive diameter to confirm the presence of myogenic tone.

### 2.4 Wire myography

Arterial segments (~2 mm) were mounted between two gold‐plated tungsten wires (20 μm diameter) in a Mulvany–Halpern myograph (model 400A; J.P. Trading). Changes in isometric tension were recorded via a PowerLab acquisition system using Chart 5.0 software (AD Instruments). Tissues were maintained in Krebs' buffer gassed with 95% O_2_/5% CO_2_ at 37°C and were set to a predetermined optimal resting tension of 5 mN(Narang et al., 2014). Endothelial function was assessed in each tissue by application of phenylephrine (3 μM) to induce a stable increase in tone (75% of maximal) followed by acetylcholine (3 μM). Tissues in which relaxation to acetylcholine was >90% of induced tone were deemed to have an intact endothelium. Cumulative concentration‐response curves to acetylcholine, SNP, and adenosine were constructed in arteries in which tone was raised to 75% of maximum with phenylephrine (3 μM) and relaxations expressed as a percentage of induced tone. Cumulative concentration–response curves to phenylephrine and 5‐HT were constructed in unstimulated arterial segments and expressed as a percentage of maximum response.

### 2.5 Gene expression

Second‐order mesenteric vessels were dissected and stabilized in Trizol. RNA was extracted using chloroform phase separation. A quantity of 200 μL chloroform was added per ml of Trizol and briefly vortexed before centrifugation at 12,000×*g* for 15 min at 4°C. The upper phase was collected and RNA extracted using the Qiagen RNeasy Microkit, as per manufacturer's protocol. Total RNA content and purity was determined using a Nanodrop 2000 spectrophotometer (Life Technologies Inc.). First‐strand complementary DNA (cDNA) was synthesized from 1 μg of total RNA using M‐MLV reverse transcriptase (Invitrogen). qPCR was performed with the PowerUp™ SYBR™ Green Master Mix (Applied Biosystems), using a Roche Light Cycler System (Cardiovascular Research Centre). Samples were heated to 50°C for 2 min, 95°C for 2 min, followed by 45 cycles of 15 s at 95°C, and 60 s at 60°C, 95°C for 15 min, and a temperature ramp from 60–95°C for a melting curve analysis to confirm product specificity. Gene expression was normalized to glyceraldehyde 3‐phosphate dehydrogenase (GAPDH) and analyzed relative to expression in the WT mice. Oligonucleotide primers used are shown in Table 1.

**TABLE 1:** qPCR primers

*Gene symbol*	*Primer Sequence (5’ ‐ 3’)*	*Accession number*
*Slc29a1*	F: CCACCAACAGAAACCAGTCTAT R: ACCCAATGGTAACCGTGAAG	NM_001199113.1
*Slc29a2*	F: ATCCTCCTCTCCATCGTATGT R: CTTGGAGGAGCTCAGCTTTAG	NM_007854.3
*Slc29a4*	F: CAGGGACCTCCATCGTATTTG R: TTCAACCTCTCCACCACAAC	NM_146257.2
*Slc22a1*	F: CAACCTCTACCTGGACTTCTTT R: CCAGATTTGATGCCGCTATTG	NM_009202.5
*Slc22a2*	F: GTCCTTGTCTGCTCCTCTATG R: GGCCAACCACAGCAAATAC	NM_013667.3
*Slc22a3*	F: CACCCTCGGGATCATTATTCTT R: TCAGGGACCACCCAGTAATA	NM_011395.2
*Slc28a2*	F: GATTGCCTTTCTGGCTGTATTG R: GCAGATGACCTGGAAACTGA	NM_172980.3
*Slc6a4*	F: CCCTCTGTTTCTCCTGTTCATC R: GCAGTAGCCCAAGATGATACTC	NM_010484.2
*Htr1a*	F: CTGTTTATCGCCCTGGATGT R: CGTCCTCTTGTTCACGTAGTC	NM_008308.4
*Htr1b*	F: CCAAAGCAGAGGAGGAGATG R: GAGCAGGGTGGGTAAATAGAAA	NM_010482.2
*Htr1d*	F: CGTCCTTACCACCATTCTACTC R: CCAAGATAGAAACCAGGAGGTC	NM_001285482.1
*Htr1f*	F: CACCACCCAGCCAACTATTTA R: CTTGTCCCATAATCCAGCTCTC	NM_008310.3
*Htr2a*	F: CACCATTGCGGGAAACA R: AGGAAACCCAGCAGCATATC	NM_172812.3
*Htr2b*	F: CCTGATACTCGCGGTGATAATAC R: CTGCTATCGCCAAGGACATTA	NM_008311.3
*Htr4*	F: GCCTTCTACATCCCGTTTCTC R: GCCCGTTGTAACATCTGTATCT	NM_008313.4
*Htr7*	F: GCAGCCAAACACAAGTTCTC R: ACACTCTTCCACCTCCTTCT	NM_008315.3
*Adora1*	F: CCCTCATCCTCTTCCTCTTTG R: GATGAGGATGCTGGGTTTCT	NM_001039510.2
*Adora2a*	F: CTCACGCAGAGTTCCATCTT R: CCGTCACCAAGCCATTGTA	NM_009630.3
*Adora2b*	F: CTCACACAGAGCTCCATCTTTAG R: GTCCCAGTGACCAAACCTTTA	NM_007413.4
*Adora3*	F: GCCATTGCTGTAGACCGATAC R: CCAGCAAAGGCCCAAGAATA	NM_009631.4
*Adk*	F: GATGGCCGTCATGCCTTAT R: CCTGCGTCTTTCTGGCTATT	U26589.1
*Ada*	F: GAGGTGTTGGAGCTGTGTAAG R: GGCTACTTCCTTCAATGGTCTC	NM_001272052.1
*Maoa*	F: AAGAACCACAGGGCAGATAC R: GCTGAGGAATGGGACAAGATAA	NM_173740.3
*Maob*	F: GGGACTACATGACAATGAAAGA R: CTCCACACTGCTTCACATAC	NM_172778.2
*Tph1*	F: GACCATCTTCCGAGAGCTAAAC R: CTTCCCGATAGCCACAGTATTT	NM_009414.3
*Entpd1*	F: TCTCTCTCCTGCAAGGCTATAA R: TCAGCATGTAGCCCAAAGTC	NM_001304721.1
*Nt5e*	F: GGTGTGGAAGGACTGATTGAT R: CCGCCAACAGAGAGAACTTTA	NM_011851.4
*Nos1*	F: AAGAGGAGAGGAAGAGCTACAA R: CAAAGTTGTCTCTGAGGTCTGG	NM_008712.3
*Nos2*	F: CTTTGACGCTCGGAACTGTA R: GACCTGATGTTGCCATTGTTG	NM_010927.4
*Nos3*	F: GGATGAGTATGATGTGGTGTCC R: CTGCAAAGCTCTCTCCATTCT	NM_008713.4
*Cox1*	F: GGATACTGGCTCTGGGAATTTG R: GTAGTCATGCGCTGAGTTGTAG	NM_008969.4
*Cox2*	F: GTGCCTGGTCTGATGATGTATG R: TGAGTCTGCTGGTTTGGAATAG	NM_011198.4
*Ptgis*	F: GATGGGAAACGGCTGAAGAA R: GGTCGAAATGAGTCAGCAGTAG	NM_008968.4
*Ptges*	F: GCAACGACATGGAGACAATCTA R: TGTGAGGACAACGAGGAAATG	NM_022415.3
*Ptges2*	F: CATTAGTGCCCTCAAGACCTAC R: CCTTGCCCTGGTCATTCAT	NM_133783.2
*Gapdh*	F: GGGTGTGAACCACGAGAAATA R: GTCATGAGCCCTTCCACAAT	NM_001289726.1

### 2.6 Drugs

All chemicals were purchased from Sigma‐Aldrich apart from 1‐[(2 chlorophenyl) diphenylmethyl]‐1H‐pyrazole (TRAM‐34) and apamin which were from Tocris. For the isolated artery experiments, drugs were dissolved in Krebs' buffer except for TRAM‐34 which was dissolved as a stock solution in DMSO before being diluted into Krebs' buffer.

### 2.7 Data analysis

All values are shown as mean ± *SEM* for N independent experiments from different mice. Statistical differences between mean values were determined using Student's *t* test for multiple comparisons or two‐way ANOVA with Tukey's multiple comparisons posttest, as appropriate to experimental design, using the statistical analysis functions of GraphPad Prism 8.2. Differences in parameters derived from multiple curve fits (e.g., logEC_50_ values) were also assessed using GraphPad Prism 8.2 based on the Extra sum‐of‐squares *F* test (*p* < .05).

## 3 RESULTS

**FIGURE 1:**
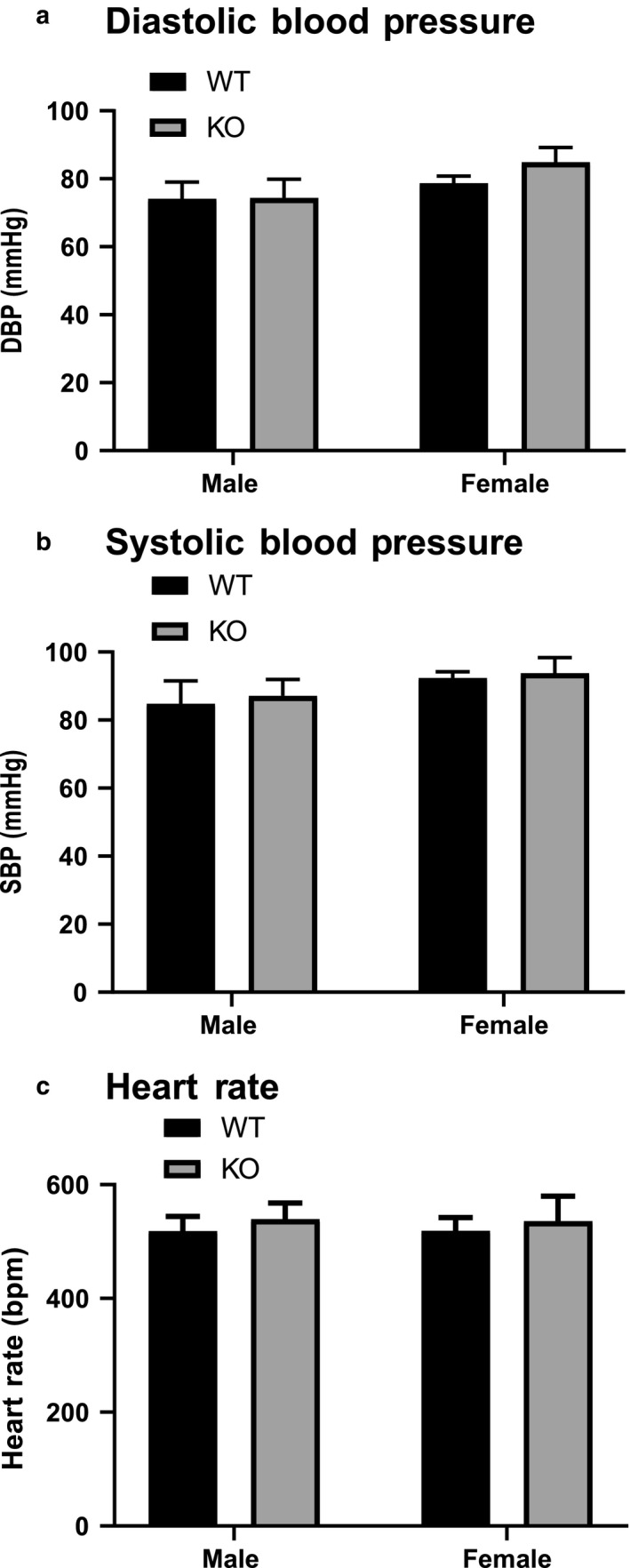
‘Hemodynamic characteristics of male and female WT and ENT4‐KO mice. Diastolic (a), systolic (b), and heart rate (c) were assessed via a pressure sensor inserted into the left carotid artery of anesthetized mice. Data are shown as mean ± *SEM* from 11 male WT, 13 female WT, 10 male ENT4‐KO, and 9 female ENT4‐KO mice. Data were analyzed using two‐way ANOVA with multiple comparisons assessed using the Bonferroni posttest (*p* < .05). There were no significant differences between groups for any of the parameters tested

The ENT4‐KO mice and WT mice had a similar gross morphology. The ENT4‐KO mice had no reproductive issues with similar litter size and frequency as the WT mice. There were also no genotype differences in diastolic or systolic blood pressure or heart rate (Figure 1).

### 3.1 Superior mesenteric artery flow rates

**FIGURE 2:**
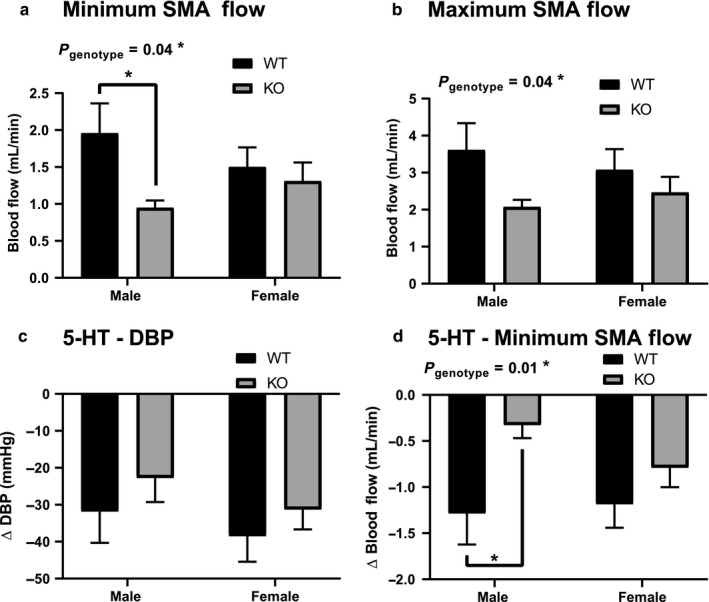
Decrease in superior mesenteric artery blood flow in male ENT4‐KO mice, and the effect of 5‐HT. Minimum (a) and maximum (b) blood flow was measured in the superior mesenteric artery (SMA) in anesthetized male and female WT and ENT4‐KO mice. The net decrease in diastolic blood pressure (c) and minimum SMA flow rate (d) was assessed after application of a bolus dose of 50 µg/kg 5‐HT. Each bar represents the mean ± *SEM* from 7 mice. *denotes a significant difference between the indicated groups (2‐way ANOVA with Bonferroni multiple comparison test, *p* < .05)

Minimum blood flow through the superior mesenteric artery was reduced significantly in the male ENT4‐KO mice (0.95 ± 0.10 mL/min), to about half of that seen in male WT mice (1.96 ± 0.40 mL/min) (Figure 2a). There was also an overall genotype‐linked significant difference (decrease in ENT4‐KO mice) in the maximum blood flow with the majority of the effect occurring in the male mice (Figure 2b). A dose of 5‐HT (50 µg/kg) that had no significant effect on diastolic (Figure 2c) or systolic blood pressure or heart rate, reduced blood flow through the superior mesenteric artery in both male and female WT mice by about 1.2 mL/min (Figure 2d). The effect of 5‐HT on mesenteric flow in the male ENT4‐KO mice was reduced significantly, to 25% (−0.33 ± 0.14 mL/min) of that seen for the male WT mice (−1.29 ± 0.34 mL/min) (Figure 2d). In contrast, there was no difference between female WT and ENT4‐KO mice in this regard.

**FIGURE 3:**
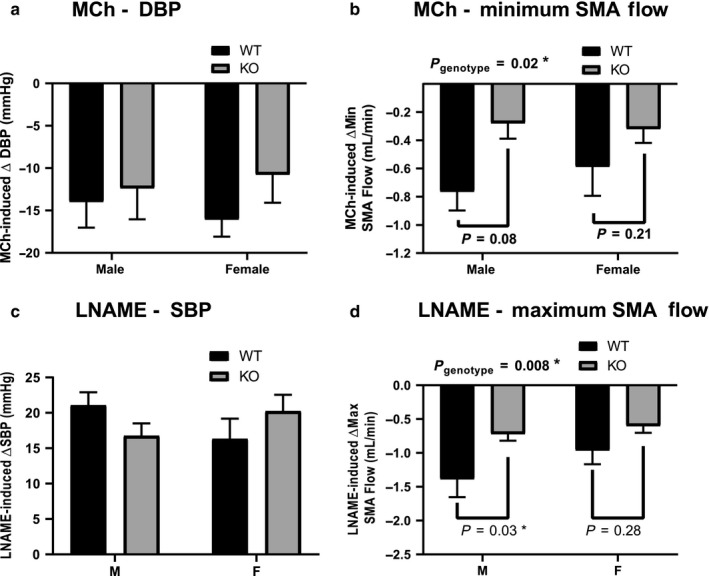
Effect of methacholine and L‐NAME on blood pressure and superior mesenteric artery blood flow WT and ENT4‐KO mice. Mice were given a bolus dose of either 1 µg/kg methacholine (MCh; a, b) or 60 mg/kg L‐NAME (c, d) and blood pressure and superior mesenteric artery (SMA) blood flow was measured. Each bar represents the mean ± *SEM* from 6 male WT, 8 female WT, 7 male ENT4‐KO, and 7 female ENT4‐KO mice. *Significant difference between the indicated groups (two‐way ANOVA with Bonferroni multiple comparison test, *p* < .05)

**FIGURE 4:**
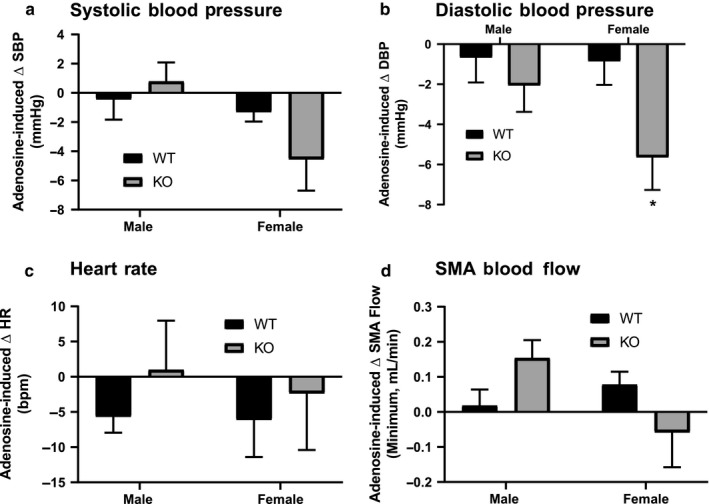
Effect of adenosine on blood pressure, heart rate, and mesenteric blood flow in WT and ENT4‐KO mice. Mice were given a bolus dose of 100 mg/kg adenosine and the net change in blood pressure (a, b), heart rate (c) and minimum blood flow (d) through the superior mesenteric artery (SMA) was assessed. Each bar represents the mean ± *SEM* from 6 male WT, 7 female WT, 7 male ENT4‐KO, and 5 female ENT4‐KO mice. *Significant difference between WT and ENT4‐KO response (2‐way ANOVA with Bonferroni multiple comparison test, *p* < .05)

To determine whether these sex‐dependent differences reflected changes in endothelial‐mediated regulation of vascular reactivity, we assessed the effect of the endothelial‐dependant vasodilator methacholine and the NO synthase (NOS) inhibitor L‐NAME. Methacholine caused a significant decrease in diastolic blood pressure, with no significant difference between male and female mice, nor between genotypes (Figure 3a). Methacholine also decreased blood flow through the superior mesenteric artery of the WT mice but had significantly less effect on mesenteric flow in the ENT4‐KO mice (Figure 3b). L‐NAME caused an increase in systolic blood pressure with no differences between sexes or genotype (Figure 3c). This same dose of L‐NAME caused a significant decrease in maximum mesenteric blood flow in the WT mice, but this effect was attenuated significantly in the ENT4‐KO mice, particularly in the males (Figure 3d). Bolus administration of 100 mg/kg adenosine had no significant effect on diastolic or systolic blood pressure, heart rate, or mesenteric artery flow rates in the WT mice (Figure 4). Adenosine did, however, induce a statistically significant decrease in diastolic blood pressure in female ENT4‐KO mice (Figure 4b). There was a trend toward an increase in mesenteric artery flow in the male ENT4‐KO mice relative to that seen in male WT mice, but it did not reach statistical significance in this study (Figure 4d).

### 3.2 Myogenic reactivity in isolated mesenteric resistance arteries

**FIGURE 5:**
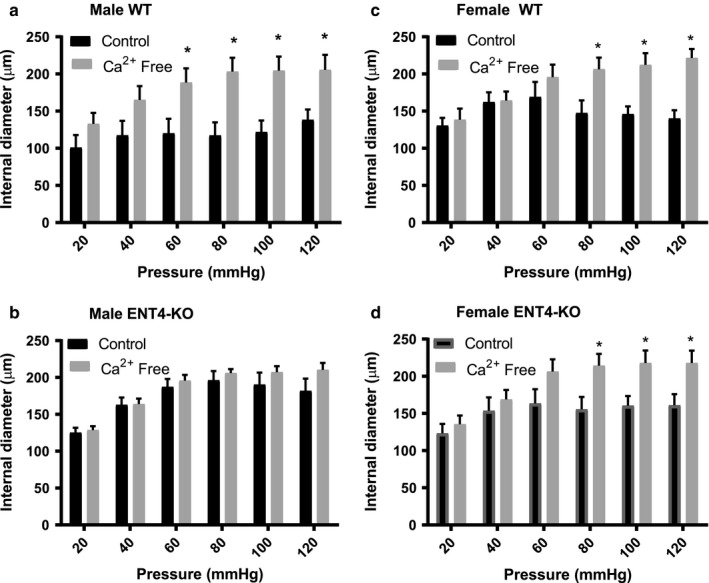
Loss of myogenic tone in mesenteric arteries of male ENT4‐KO mice. Second‐order mesenteric arteries isolated from male WT (a) and ENT4‐KO (b) mice and female WT (c) and ENT4‐KO (d) mice were subjected step‐wise to increased intravascular pressures between 20 and 120 mmHg, and the internal diameters measured. Pressure ramps were conducted in both normal Kreb's media (Control), and Ca^2+^‐free media (Ca^2+^ Free). Each bar represents the mean ± *SEM* from five experiments. *Significant difference in internal diameter between control and Ca^2+^‐free conditions (Student’s *t* test, *p* < .05)

Mesenteric arteries from male and female WT mice developed myogenic tone in response to increases in intravascular pressure above 60 and 80 mmHg, respectively (Figure 5a & c). This tone was completely absent in arteries from male ENT4‐KO mice (Figure 5b), while arteries from female ENT4‐KO mice exhibited a myogenic response that was similar to that of WT mice (Figure 5d). The maximum passive diameters of the vessels were not different between male and female and WT and ENT4‐KO mice (~ 210 µm internal diameter under Ca^2+^‐free conditions at 120 mmHg pressure).

### 3.3 Phenylephrine‐induced increases in tone and acetylcholine‐ and SNP‐evoked relaxations in mesenteric resistance arteries

**FIGURE 6:**
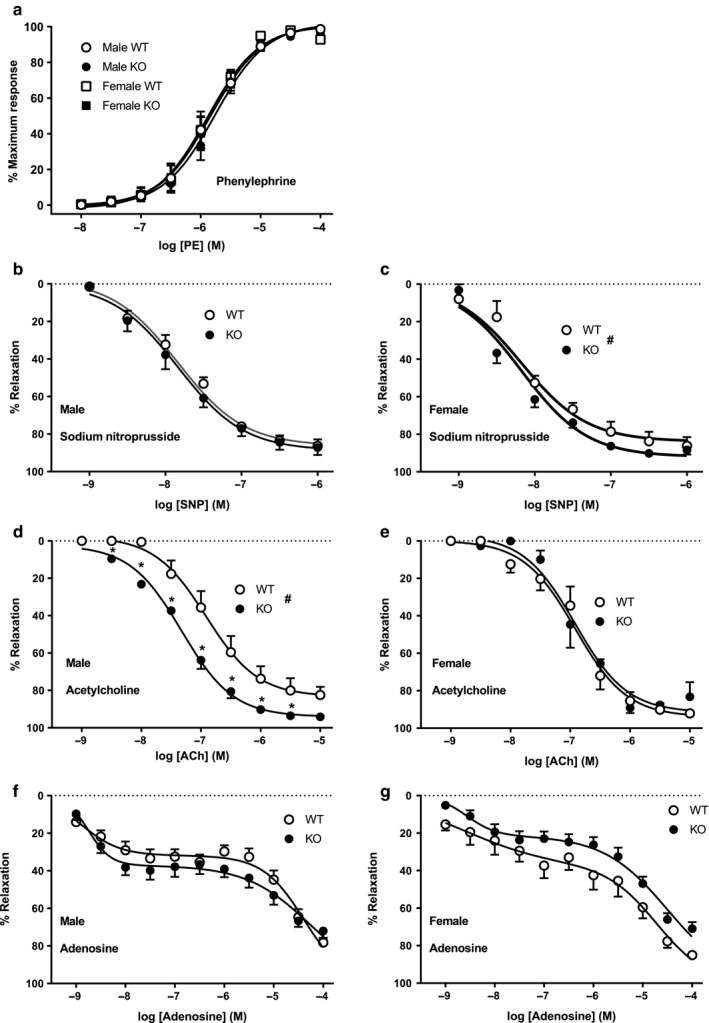
Responsiveness of mesenteric resistance arteries to phenylephrine, acetylcholine, SNP, and adenosine. Mesenteric artery rings were isolated from male and female WT and ENT4‐KO mice and mounted in a myograph under a resting tension of 5 mN. Panel (a) shows the concentration‐dependent increases in tone evoked by cumulative concentrations of phenylephrine. Panels (b–g) indicate the relaxation of prestimulated mesenteric rings to cumulative concentrations of SNP (b, c), acetylcholine (d, e), and adenosine (f, g) in male and female mice. Data are presented as % of maximum response, and shown as mean ± *SEM* from 5 independent experiments. *Significant difference between WT and ENT4‐KO response (Student's *t* test, *p* < .05). ^#^Significant difference in logEC_50_ calculated from curve fits of the WT and ENT4‐KO data (Akaike's information criteria method, GraphPad Prism v 8.2)

There were no differences between any of the groups for phenylephrine‐induced increases in tone (logEC_50_ ~ −5.8; Figure 6a). Likewise, there was no difference between genotypes in their response to the endothelium‐independent vasorelaxant SNP (Figure 6b & c), although arteries from female mice (WT logEC_50_ = −8.07 ± 0.09) were slightly more sensitive to SNP than were those from male mice (WT logEC_50_ = −7.75 ± 0.08) (Figure 6c). The response to the endothelium‐dependent relaxant acetylcholine, on the other hand, differed significantly between arteries isolated from male WT versus male ENT4‐KO mice (logEC_50_ of −6.91 ± 0.11 and −7.33 ± 0.05 for the WT and ENT4‐KO arteries, respectively; Figure 6d). Arteries from female mice showed no differences (WT versus KO) in their sensitivity to acetylcholine (Figure 6e), with logEC_50_ values similar to those seen for arteries from male WT mice.

### 3.4 Adenosine‐evoked relaxation of mesenteric resistance arteries

Adenosine induced a significant biphasic relaxation of preconstricted mesenteric arteries (Figure 6f,g). In arteries from WT mice, adenosine reduced tone by about 35% with an EC_50_ of ~ 2 nM (logEC_50_ of −8.87 ± 0.17 and −8.61 ± 0.81 for male and female mice, respectively), with no significant difference between sexes. Higher concentrations of adenosine induced further relaxation, with arteries from female WT mice having a significantly greater sensitivity to adenosine for this lower‐affinity (EC_50_ ~ 30 µM) component compared with those from male WT mice (logEC_50_ of −4.39 ± 0.08 and −4.72 ± 0.27 for male and female mice, respectively). Arteries from male ENT4‐KO mice were similar to those from male WT mice with respect to their response to adenosine (~35% high affinity component, and logEC_50_ values of −8.75 ± 0.11 and −4.27 ± 0.14 for the high and low affinity components, respectively) (Figure 6f). Likewise arteries from female ENT4‐KO mice were similar to those from female WT mice in terms of the EC_50_ of adenosine for the high and low affinity adenosine‐mediated relaxation components (logEC_50_ of −8.57 ± 0.21 and −4.51 ± 0.10, respectively) (Figure 6g). However, arteries from the female ENT4‐KO mice had significantly less of the high affinity adenosine‐mediated relaxation component compared to those from female WT mice (21 ± 3% versus 37 ± 15% for ENT4‐KO and WT, respectively).

### 3.5 Contribution of NOS and K_Ca_ channels to endothelium‐dependent relaxation by acetylcholine in mesenteric resistance arteries

**FIGURE 7:**
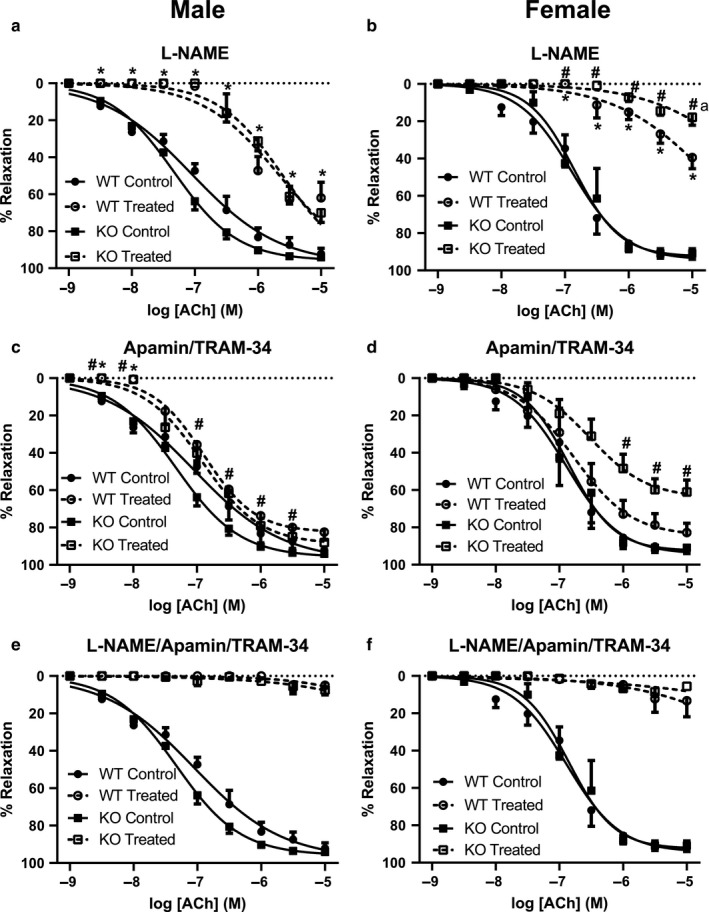
Contribution of NO and K_Ca_ channels to the relaxant effect of acetylcholine in isolated mesenteric artery rings. Acetylcholine dose–response curves were generated, as described for Figure 6, in the absence (Control) and presence (Treated) of L‐NAME (to inhibit NO production) (a, b), TRAM‐34 + apamin (to inhibit K_Ca_ channels) (c, d), or L‐NAME + TRAM‐34 + apamin (to inhibit both NO and K_Ca_) (e, f). Each point represents the mean ± *SEM* obtained using five male WT, nine male ENT4‐KO, six female WT, and nine female ENT4‐KO arteries. Differences were assessed for significance by 2‐way ANOVA followed by the Bonferroni posttest for multiple comparisons with *p* < .05 considered significant. *Significantly different from Control. ^#^Significant difference between WT and ENT4‐KO response to treatment

Acetylcholine concentration–response curves were generated in the absence and presence of the NOS inhibitor L‐NAME (100 µM), the intermediate conductance K_Ca_ (IK_Ca_) channel inhibitor TRAM‐34 (1 µM) + the small conductance K_Ca_ (SK_Ca_) channel inhibitor apamin (50 nM), or L‐NAME + TRAM‐34 + apamin. L‐NAME had a significant inhibitory effect on acetylcholine‐evoked relaxation in all groups. However, it had a slightly greater effect on the relaxations in arteries from female (WT: logEC_50_= −4.72 ± 0.13; ENT4‐KO: logEC_50_ = −3.91 ± 0.09) relative to male mice (WT: logEC_50_: −5.66 ± 0.09; ENT4‐KO: logEC_50_ = −5.61 ± 0.05) (Figure 7a,b). K_Ca_ channel block with apamin/TRAM‐34 had minimal effect on acetylcholine‐induced relaxation in arteries from the male WT mice (Figure 7c), with a significant effect seen only at low concentrations of acetylcholine. However, K_Ca_ channel blockade effectively reversed the enhanced sensitivity of the arteries from male ENT4‐KO mice to acetylcholine, such that in the presence of apamin/TRAM‐34, there was no difference in the acetylcholine response between the male WT and ENT4‐KO mice (logEC_50_ of −6.58 ± 0.08 and −6.73 ± 0.05, respectively). Arteries from female WT mice were similar to those from male WT mice in that apamin/TRAM‐34 had no significant effect on the acetylcholine‐induced relaxation (Figure 7d). However, arteries from female ENT4‐KO mice were significantly more sensitive to K_Ca_ blockade than that seen for the WT mice (49 ± 8% and 73 ± 7% relaxation to 1 μM acetylcholine in the presence of TRAM‐34/apamin in the female ENT4‐KO and WT tissues, respectively; Figure 7d). The combination of L‐NAME and TRAM‐34/apamin blocked all acetylcholine‐induced relaxation in arteries regardless of sex or genotype (Figure 7e,f).

### 3.6 Effect of 5‐HT on mesenteric resistance arteries

**FIGURE 8:**
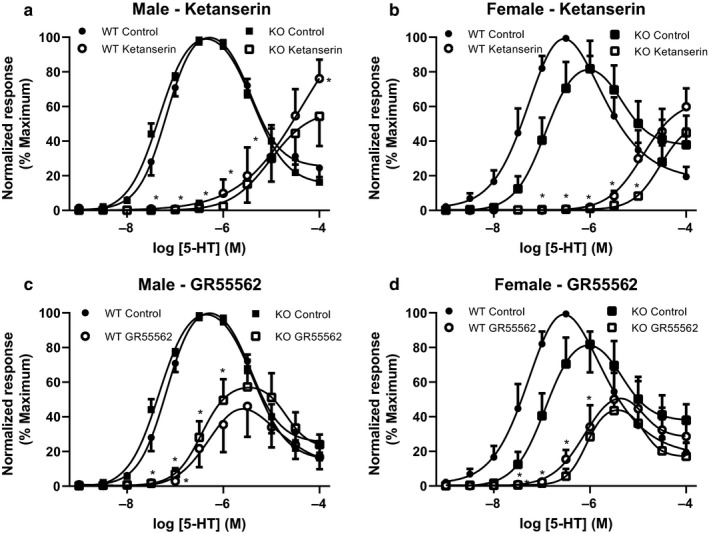
Effect of 5‐HT receptor antagonists on the response of mesenteric artery rings to 5‐HT. Mesenteric artery rings from male (a, c) and female (b, d) WT and ENT4‐KO mice were exposed to increasing concentrations of 5‐HT and the degree of vascular contraction shown as the % of maximum response of the tissue. The response to 5‐HT was assessed in the absence (Control) and presence of the 5‐HT_2A_/5‐HT_1B/1D_ receptor blocker ketanserin (30 nM) (a, b), or the 5‐HT_1B/1D_ receptor blocker GR55562 (1 µM) (c, d). Each point represents the mean ± *SEM* from either 4 (a, c) or 5 (b, d) experiments. Differences were assessed for significance by two‐way ANOVA followed by the Bonferroni posttest for multiple comparisons with *p* < .05 considered significant. *Significantly different from control

5‐HT induced a biphasic response in mesenteric arteries, with an increase in tone observed at concentrations up to ~ 1 µM and then a relaxation at higher concentrations (Figure 8). There was no significant difference in the response to 5‐HT in arteries from male WT and ENT4‐KO mice (Figure 8a). However, in this data set, arteries from female ENT4‐KO mice displayed a significantly reduced sensitivity to 5‐HT as compared to arteries from female WT mice (45 ± 19% and 16 ± 5% of maximum contraction by 30 nM 5‐HT for the WT and ENT4‐KO, respectively; Figure 8b). The relaxant effect at higher concentrations of 5‐HT was similarly shifted to the right in arteries from the female ENT4‐KO mice.

In the presence of the 5‐HT_2A_ antagonist ketanserin (30 nM), 5‐HT produced only a weak constricting effect in arteries from WT and ENT4‐KO male and female mice (Figure 8a,b). The 5‐HT_1B/1D_ receptor blocker GR55562 (1 μM) significantly reduced 5‐HT‐evoked increases in tone in arteries from mice of both sexes and genotypes, but had no effect on the relaxant effect of higher concentrations of 5‐HT (Figure 8c,d). There were no differences between groups in the effects of either ketanserin or GR55562.

**FIGURE 9:**
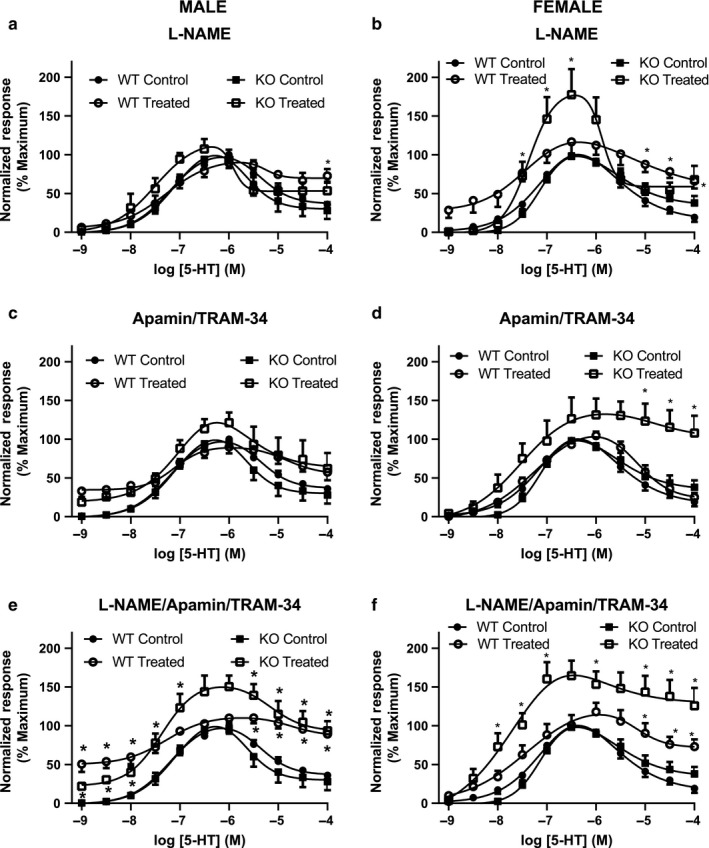
Contribution of NO and K_Ca_ channels to the effects of 5‐HT in isolated mesenteric artery rings. Concentration response curves were generated, as described for Figure 7, in the absence (Control) and presence (Treated) of L‐NAME (to inhibit NO production) (a, b), TRAM‐34 + apamin (to inhibit K_Ca_ channels) (c, d), or L‐NAME + TRAM‐34 + apamin (to inhibit both NO and K_Ca_) (e, f). Each point represents the mean ± *SEM* from 5 (a, b, c) or 6 (d, e, f) experiments. Differences were assessed for significance by two‐way ANOVA followed by the Bonferroni posttest for multiple comparisons with *p* < .05 considered significant. *Significantly different from Control

To assess the role of NO and/or K_Ca_ channels in endothelial modulation of the response of mesenteric arteries to 5‐HT, concentration–response profiles were constructed in the presence and absence of the NOS inhibitor L‐NAME or the K_Ca_ channel blockers apamin/TRAM‐34, as described above for acetylcholine (see Figure 7). L‐NAME had no effect on the 5‐HT responses in arteries from male WT mice (Figure 9a). L‐NAME did, however, slightly enhance the contraction caused by 5‐HT in arteries from the female WT mice, and dramatically increased the maximum 5‐HT response in arteries from female ENT4‐KO mice (Figure 9b). Like that seen for L‐NAME, apamin/TRAM‐34 had no significant effect on the 5‐HT response in arteries from male WT or ENT4‐KO mice (Figure 9c). Arteries from female WT mice also had a similar response to 5‐HT in the presence and absence of apamin/TRAM‐34 (Figure 9d). However, in arteries from female ENT4‐KO mice, K_Ca_ channel blockade completely prevented the relaxation phase of the 5‐HT response (Figure 9d). Application of the combination of L‐NAME, TRAM‐34 and apamin led to significantly greater responses to 5‐HT in arteries from both male and female mice, with the greatest effect seen in the ENT4‐KO groups (Figure 9e,f).

### 3.7 Gene expression in mesenteric arteries

**FIGURE 10:**
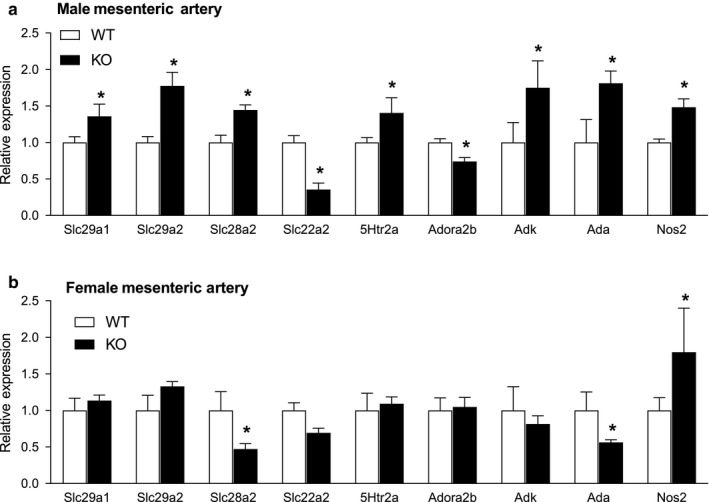
Impact of the loss of ENT4/*slc29a4* on the expression of the nucleoside transporters ENT1 (*slc29a1*), ENT2 (*slc29a2*), CNT2 (*slc28a2*), as well as 5‐HT_2a_ receptor (*5htr2a*) the organic cation transporter OCT2 (*slc22a2*), adenosine kinase (*adk*), adenosine deaminase (*ada*), and iNOS (*nos2*). mRNA was isolated from the mesenteric arteries of male (a) and female (b) WT and ENT4‐KO mice. Quantitative PCR was used to assess the relative (to WT) gene expression using GAPDH as the internal reference. Each bar represents the mean ± *SEM* from six experiments. *Significant difference between WT and ENT4‐KO (Student's *t* test, *p* < .05)

There was no difference in ENT4 expression in arteries from male and female WT mice (∆Ct of 10.4 ± 0.8 and 10.1 ± 0.6 for male and female, respectively, *N* = 6). *Nos2*, encoding for iNOS, was significantly upregulated in mesenteric arteries from both male and female ENT4‐KO mice relative to WT (Figure 10). *Slc28a2*, which encodes for a purine selective concentrative nucleoside transporter CNT2, was upregulated in mesenteric arteries from male ENT4‐KO mice but down‐regulated in those from female ENT4‐KO mice. *Ada* (encoding adenosine deaminase) was also downregulated in arteries from female ENT4‐KO mice but upregulated in arteries from male ENT4‐KO mice. Arteries from male ENT4‐KO mice showed additional changes in gene expression that were not observed in the female ENT4‐KO mice. Specifically, *slc29a1* and *slc29a2*, encoding the equilibrative nucleoside transporters ENT1 and ENT2, respectively, were significantly upregulated in arteries from the ENT4‐KO male mice. Likewise, *adk* (encoding adenosine kinase) was upregulated in arteries from the ENT4‐KO males (in parallel with *ada* as noted above). Mesenteric arteries from male ENT4‐KO mice also showed significant downregulation of *slc22a2* (OCT2) and a*dora2b* (adenosine A2b receptor), and an upregulation of *5htr2a* (5‐HT2a receptor). There were no differences between genotypes for any of the other genes tested (transporters: *slc22a1*/OCT1, *slc22a3*/OCT3, *slc6a4*/SERT; 5HT receptors *htr1a*, *htr1b*, *htr1d*, *htr1f*, *htr2b*, *htr4*, *htr7*; adenosine receptors: *adora1*, *adora2b*, *adora3*; enzymes: *entpd1*, *nt5e*, *maoa*, *maob*, *tph1*, *nos1*, *nos3*, *cox1*, *cox2*, *ptgis*, *ptges*, *ptges2*).

## 4 DISCUSSION

Regulation of vascular tone involves a complex interplay of a variety of endogenous relaxant and constricting factors that are released under various physiological and pathophysiological circumstances from platelets, endothelial cells, and vascular smooth muscle (Gheibi, Jeddi, Kashfi, & Ghasemi, 2018; Jackson, 2018; Loh et al., 2018). The ENT4 substrates adenosine and 5‐HT are prominent among these endogenous regulators. Dynamic regulation of local blood flow is generally attributed to vascular endothelial mechanisms, specifically NOS generated NO and K_Ca_ channel‐mediated endothelial hyperpolarization (EDH; Kerr et al., 2012; Khaddaj Mallat, 2017). Thus, we assessed the relative contribution of NO and EDH mechanisms to the responses of isolated mesenteric arteries to the vascular regulators acetylcholine/methacholine, adenosine, and 5‐HT in both male and female WT and ENT4‐KO mice. These agents were also tested in vivo for their effects on overall hemodynamics and mesenteric blood flow in these mice. The data obtained highlight distinct differences in vascular regulation between male and female WT mice, as well as significant differences in how the vasculature of male and female mice respond to the loss of ENT4.

### 4.1 Male/female comparison

Myogenic tone was lower in female mesenteric arteries relative to male arteries, consistent with what has been reported previously (Veerareddy, Cooke, Baker, & Davidge, 2004). A dichotomy was also apparent with respect to the effect of 5‐HT on isolated mesenteric arteries. Our results suggest that mesenteric arteries from female mice are modulated by NO to a greater extent than are arteries from male mice. This implies that factors that decrease NO activity in the vasculature, such as that which occurs in endothelial dysfunction, would impact 5‐HT‐mediated regulation in females more than males (at least with respect to the mouse mesenteric artery). Relatively few studies have examined sex differences in the response to 5‐HT, with many of the studies on the vascular effects of 5‐HT in mice being conducted using male animals only. It has been reported that endothelium‐intact human internal mammary artery segments from women exhibit increased sensitivity to the contractile actions of 5‐HT compared to those from men (Lamin et al., 2018). It was established that this difference was due to enhanced contribution of the endothelial cyclooxygenase pathway to the 5‐HT effects in males versus females. There is also a sex difference in the CNS‐mediated effects of 5‐HT on cardiovascular function; Magnusson and colleagues showed that in male rats, central 5‐HT mechanisms facilitate sympathetic vascular tone, while in females 5‐HT reduces cardiac vagal activity (Magnusson & Cummings, 2017). Our data add to this body of literature describing significant differences in vascular regulation between males and females, and highlight the importance of including both sexes in any research studies on vascular function.

### 4.2 Effect of 5‐HT

5‐HT had a biphasic effect on mesenteric arteries, where low concentrations (10–300 nM) of 5‐HT‐induced contraction of the arteries, while a relaxation was evident at concentrations greater than 1 µM. The contraction phase was likely mediated by 5‐HT_2A_ receptors on vascular smooth muscle which are coupled to L‐type Ca^2+^ channels, phospholipase C and tyrosine kinases (McKune & Watts, 2001; Watts, 2002). Our data showing that ketanserin, a 5‐HT_2A_ receptor blocker, prevented the contractile effect of 5‐HT supports this supposition. However, the contractile effect of 5‐HT was also inhibited by the selective 5‐HT_1B/1D_ receptor blocker GR55562, albeit not as effectively as by ketanserin. 5‐HT_1B/1D_ receptors in smooth muscles cells have been reported to mediate vasoconstriction in rat cerebral artery (Kovacs, Harsing, & Szenasi, 2012), bovine pulmonary artery (McKenzie, Alapati, Macdonald, & Shaw, 2010), rabbit renal artery (Hill, Dora, Hughes, & Garland, 2000) and guinea pig iliac artery (Jahnichen, Radtke, & Pertz, 2004). This suggests that 5‐HT_1B/1D_ receptor stimulation also contributes to the 5‐HT‐evoked tone in mouse mesenteric arteries, possibly through ROK‐mediated Ca^2+^‐sensitization mechanisms (Nuno, Korovkina, England, & Lamping, 2007). The relaxation of the mesenteric arteries at the higher concentrations of 5‐HT was likely mediated by 5‐HT_2B_ and/or 5‐HT_7_ receptors (Chang Chien, 2015).

### 4.3 Effect of adenosine

Arteries from male and female WT mice responded similarly with a biphasic relaxant response to adenosine. Adenosine mediates a vasodilatory response in resistance vessels through interactions with vascular adenosine A2 receptors via a mechanism involving NO. There are two subtypes of adenosine A2 receptors, A2a and A2b, that differ in their affinity for adenosine with the adenosine A2b receptor having an ~50‐fold lower affinity for adenosine (~15 µM) relative to the A2a receptor (~300 nM; Fredholm, Ijzerman, Jacobson, Linden, & Müller, 2011). Based on the EC_50_ values obtained in the present study, the relaxation seen with adenosine concentrations of less than 1 µM was likely mediated by the adenosine A2a receptor, while the second phase of relaxation observed with higher adenosine concentrations was likely mediated by the adenosine A2b receptor (Borea, Gessi, Merighi, Vincenzi, & Varani, 2018). The predominance of the A2b‐mediated component to the overall effect of adenosine in murine mesenteric arteries is consistent to what has been found in other studies (Teng et al., 2013). Due to the vasodilatory response to adenosine by resistance vessels, an in vivo bolus application of adenosine typically leads to a transient decrease in blood pressure (Koeppen, Eckle, & Eltzschig, 2009; Teng, Tilley, Ledent, & Mustafa, 2016). The lack of effect of a bolus injection of 100 mg/kg adenosine on blood pressure and mesenteric artery flow in the WT mice (both male and female) in the present study was thus unexpected. It may be of interest in future studies using this mouse model to examine the effect of adenosine infusion, which has been shown to have more consistent effects on both heart rate and blood pressure in mice (Hansen et al., 2006).

### 4.4 Impact of slc29a4 (ENT4) knockout on mesenteric vascular regulation and function

The most striking observation was the complete loss of myogenic tone in the secondary mesenteric arteries of male ENT4‐KO mice. There was no apparent difference in the contractile capacity of these vessels, as arteries from both male WT and KO mice had a similar contractile response to the α_1_‐adrenoceptor agonist phenylephrine. It is interesting that there was a corresponding male‐specific decrease in blood flow in the superior mesenteric artery in the ENT4‐KO mice. This may be a physiological compensation for the dilated state of the downstream mesenteric resistance arteries due the lack of myogenic tone, to maintain homeostasis in perfusion pressure throughout the mesenteric vascular bed. The mechanism(s) underlying the male‐specific loss in tone of the mesenteric resistance vessels may be related to sex‐specific differences in both NO and K_Ca_ regulated vascular signaling. Arteries from male ENT4‐KO mice had an increased sensitivity to the NO‐dependent relaxant effect of acetylcholine, when compared with the female ENT4‐KO mice. Mesenteric arteries from female ENT4‐KO mice exhibited an enhanced (relative to WT and male ENT4‐KO mice) vasodilatory tone mediated by both K_Ca_ channels (blocked by TRAM‐34/apamin) and NO (blocked by L‐NAME), suggesting that both of these endothelium‐dependent pathways may be upregulated in female mice, specifically, with the chronic loss of ENT4. It has been reported that chronic treatment with fluoxetine, an inhibitor of the high affinity serotonin transporter (SERT), leads to the loss of intracellular Ca^2+^ stores in male rat mesenteric resistance arteries (Pereira et al., 2017). Chronic loss of 5‐HT uptake by the ENT4 transporter in mice may have similar effects on Ca^2+^ stores, which could impact vessel myogenic tone and acetylcholine (via NO) actions(Wilson, Lee, & McCarron, 2016) and thereby disrupt normal regulation of vascular function. These differences in NO and K_Ca_ channel activities in mesenteric vessels from male versus female ENT4‐KO mice are also reflected in the findings attained with 5‐HT. The maximum 5‐HT‐mediated contraction upon block of NOS and K_Ca_ channels in arteries from male and female ENT4‐KO mice was significantly greater than that seen for the arteries from the corresponding WT mice, and this difference was most pronounced in the arteries from female ENT4‐KO mice. Block of NO production in arteries from female ENT4‐KO mice enhanced the ability of low concentrations of 5‐HT to contract the vessels, while block of K_Ca_ channels enhanced the contraction seen with higher concentrations of 5‐HT. This also suggests that K_Ca_ channels, and not NO, contribute to the relaxant effects of high concentrations of 5‐HT in these arteries.

### 4.5 Compensatory changes in gene expression

A confounding consequence of a global knockout of the expression of a specific gene, as is the case for the ENT4‐KO mice, is the influence of compensatory changes in other genes that impact the system under study. iNOS was upregulated in mesenteric arteries from both male and female ENT4‐KO mice, relative to that seen in the respective WT mice. Increased iNOS expression has been correlated with vascular inflammation and endothelial dysfunction, suggesting that the vessels from the ENT4‐KO mice, regardless of sex, may be undergoing a degree of vascular stress which would disrupt NO‐mediated vascular regulation. This may underlie the increased vasoconstriction seen with 5‐HT in the mesenteric arteries from the ENT4‐KO mice. A similar enhancement in 5‐HT constrictor activity is seen in pathological conditions (e.g., diabetes, pulmonary hypertension) that result in vascular damage (Delaney et al., 2018; MacLean, 2018; Watts & Davis, 2011). With respect to other genes examined, it was the male ENT4‐KO mice that showed the most extensive changes (relative to male WT), and mostly in those genes related to adenosine metabolism (i.e., increased ADK, ADA, ENT1, ENT2, and CNT2). There were minimal changes in genes associated with 5‐HT activity or metabolism. This was unexpected, as ENT4 has been defined in the literature most prominently as a 5‐HT transporter. These data imply that loss of ENT4 is disrupting vascular adenosinergic signaling more than those involving 5‐HT, particularly in the male arteries, and supports a role for ENT4 in regulating adenosine levels in the vasculature.

## 5 CONCLUSIONS

ENT4 has been defined in the literature as a pleiotropic organic cation transporter involved mainly in the transport of monoamines. ENT4 has been reported, based on in vitro studies, to have a relatively low affinity for adenosine, with significant transport seen mainly at acidic pH (Barnes et al., 2006; Zhou et al., 2010). However, the present study suggests that ENT4 may indeed be an active contributor to the regulation of adenosine flux in vivo and contribute to both adenosine‐ and 5‐HT‐mediated regulation of vascular function in the mesentery. Unravelling the mechanisms underlying the relationship between ENT4 and NO, K_Ca_ channels, and purinergic signaling will require further investigation. Nevertheless, our study shows, for the first time, that disruption of ENT4 activity leads to significant changes in autoregulation of vascular function with potential deleterious consequences for regulation of blood flow.

## DISCLOSURE

The authors declare that they have no conflict of interest.
